# Can on-line gait training improve clinical practice? Study protocol for feasibility randomised controlled trial of an on-line educational intervention to improve clinician’s gait-related decision-making in ambulant children and young people with cerebral palsy

**DOI:** 10.1186/s40814-024-01477-5

**Published:** 2024-05-14

**Authors:** Anna Hebda-Boon, Adam P. Shortland, Aleksandra Birn-Jeffery, Dylan Morrissey

**Affiliations:** 1 Sport and Exercise Medicine, Barts and the London School of Medicine and Dentistry, Queen Mary University of London, London, UK; 2 School of Biomedical Engineering and Imaging Science, King’s College London, London, UK; 3 School of Sport, Rehabilitation and Exercises Sciences, University of Essex, Essex, UK; 4 Physiotherapy Department, Barts Health NHS Trust, London, UK

**Keywords:** Instrumented gait analysis, Gait analysis, Cerebral palsy, Physiotherapy, Paediatric physiotherapy, Educational intervention, Clinical education, Randomised controlled trial, Protocol

## Abstract

**Background:**

Instrumented gait analysis (IGA) is an assessment and research tool with proven impacts on clinical decision-making for the management of ambulant children and young people with cerebral palsy (CYPwCP) but is underused and variably understood by relevant clinicians. Clinicians’ difficulties in gaining expertise and confidence in using IGA are multifactorial and related to access for clinical decision-making, limited training opportunities and inability to translate this training into clinical practice.

**Methods:**

The primary aim of this study is to test the feasibility of an educational intervention to advance clinicians’ application of gait analysis in CYPwCP, to inform a definitive trial. The secondary aim is to measure the effect that appropriate IGA training has on physiotherapists’ knowledge, skills, confidence and behaviours. This will be a two-arm feasibility randomised controlled trial with an experimental and control group. The 6-week on-line intervention uses a multicomponent approach grounded in behavioural change techniques. A repeated measures design will be adopted, whereby participants will complete outcome measures at baseline, immediately after the intervention and at 4 months. The primary outcome measures (trial feasibility-related outcomes) are recruitment and engagement. The secondary outcome measures (trial research-related outcomes) are knowledge, skills, confidence and practice change. Outcome measures will be collected via online questionnaires and during observed skill assessments. Analysis of data will use descriptive statistics, two-way mixed ANOVA model and qualitative content analysis.

**Discussion:**

This study will determine feasibility of the definitive randomised control trial of educational intervention delivered to advance clinicians’ application of gait analysis in CYPwCP. This study offers the shift in emphasis from regarding IGA as a tool to a focus on clinicians’ requirements for access, training and a well-defined role to optimise utilisation of IGA. The impact of this should be better engagement with IGA and clinical practice change. This study will contribute to a body of educational research into clinical education of healthcare professionals and IGA training offering insight into high levels of evaluation evidence including clinical behaviour change.

**Trial registration:**

Protocol has been registered with the Open Science Framework (osf.io/nweq6) in June 2023.

**Supplementary Information:**

The online version contains supplementary material available at 10.1186/s40814-024-01477-5.

## Background

The National Institute for Clinical Excellence (NICE) refers to instrumented gait analysis (IGA) assessment as a preferable choice prior to gait-improving orthopaedic surgery [1]. The impact of IGA on decision-making in treatment planning and treatment outcomes for ambulant CYPwCP has been broadly debated in the literature particularly in areas of orthopaedic decision-making [2–5] and individually tailored nonsurgical treatments [6, 7]. Generally, single event multilevel surgeries (SEMLS) are performed after IGA is conducted as the IGA results can help to determine which specific soft-tissue or bony surgical procedures should be performed [8]. Furthermore, studies show that use of IGA for treatment decision-making has potential to improve patient outcomes — authors indicate the positive gait-related outcomes and improvement in gait parameters when treatment matches IGA recommendations [9–11]. Despite this, more standardised access pathways for CYPwCP to IGA are yet to be established [7, 12], and access to the IGA for other professionals involved in gait management such as physiotherapists or orthotists and their formal IGA education remains limited [13]. As a science, gait analysis brings a wide spectrum of knowledge and skills, making it hard to educate and successfully integrate it into undergraduate curricula [14]. Clinicians’ difficulties in gaining expertise and confidence in using IGA are multifactorial and can be related to lack of IGA access for clinical decision-making, limited training opportunities and inability to translate this training into clinical practice [15].

According to research, clinician-centred factors such as IGA training and affiliation to IGA laboratory [16] are shown to influence engagement with IGA-derived recommendations and may therefore impact on patient outcomes [17].

This indicates a required shift in emphasis from regarding IGA as a tool providing 3rd party recommendations to a focus on clinicians’ requirements for access, training and a well-defined role to optimise utilisation of IGA [17]. This is essential to address in order to improve inequity of access and patient outcomes. Findings of our previous research [15, 17] provided context for the design and delivery of a feasibility randomised controlled trial (RCT) of an educational intervention to improve clinicians’ engagement with the IGA.

### Study aims and objectives

The primary aim of this study is to determine the feasibility of an educational intervention to advance clinicians’ application of instrumented gait analysis in children and young people with cerebral palsy, to inform the design of a full trial. Objectives are as follows:

To establish the feasibility of a future randomised controlled trial of educational intervention.

1. Assess the rate of participant enrolment, retention and compliance with intervention.
2. Assess whether the inclusion and exclusion criteria for participants are appropriate.
3. Assess whether the duration of intervention is appropriate.
4. Assess whether intervention delivery in a virtual learning environment is feasible and acceptable.
5. Explore if the outcome measures are appropriate for the study aims.
6. Define the sample size for a definitive trial.
7. Explore the fidelity of intervention delivery.
8. Further understand the barriers and facilitators of the intervention.

The secondary aim is to measure the effect that appropriate IGA training and its delivery has on physiotherapists’ knowledge, skills and attitudes.

## Methods

This feasibility trial protocol follows the SPIRIT statement on defining standard protocol items for clinical trials and its checklist [18] and the CONSORT statement extension to randomised pilot and feasibility trials and its checklist [19].

### Trial design

This will be a two-arm feasibility randomised controlled trial with an experimental and control group. The 6-week on-line intervention delivered as part of the trial is a stand-alone, post-graduate level educational course called Virtual Gait Analysis Course for Paediatric Physiotherapists (VGAPP). Eligible physiotherapists who consent to take part in the study will be randomly allocated into experimental and control groups. A repeated measures design will be adopted, whereby participants will complete outcome measures at baseline, immediately after the intervention, and at 4 months. This will include collection of feedback as part of a full process evaluation.

The trial will be determined feasible if a priori set criteria based on primary outcome measures and included in the process evaluation will be achieved at or above agreed levels (see the ‘Outcome measures’ section of ‘Methods’). After conducting and reviewing outcomes of the full evaluation process, the decision about delivery of the definitive trial will be made.

**Fig. 1:**
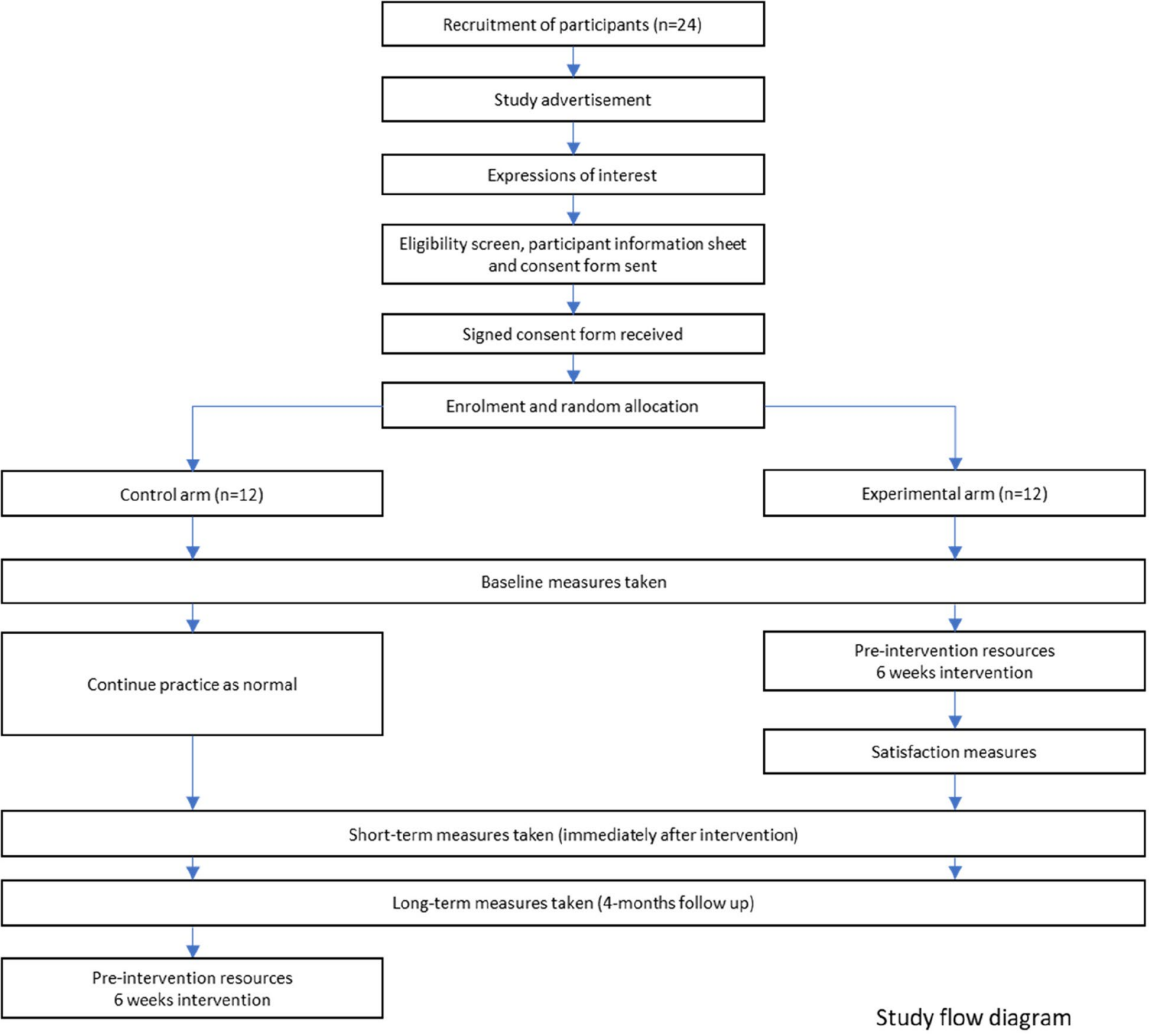
Study flow diagram

Figure 1 shows the study flow diagram, and Table 1 indicates the schedule of enrolment, intervention, and outcome measures [18].

**Table 1:** Standard Protocol Items: Recommendations for Interventional Trials (SPIRIT) diagram illustrating the study design and timescales

Design	Study period								
	**Enrolment**	**Allocation**	**Baseline**	**Pre-intervention**	**Intervention (experimental)**	**End of intervention**	**Follow-up 4 months**	**Intervention (control)**	**End of study**
**Enrolment**									
Participant recruitment	x								
Eligibility screen	x								
Informed consent	x								
Allocation		X							
**Intervention**									
Pre-intervention resources				x					
6-week intervention					x				
Live sessions					x				
**Assessments**									
Feasibility outcomes (recruitment)	x		x						
Feasibility outcomes (retention)									x
Feasibility outcomes (VLE data)						x			
Acceptability			x			x	x		
Process evaluation	x		x		x	x	x		x
Demographics			x						
Attitudes			x			x	x		
Satisfaction						x			
Self-rated knowledge			x			x	x		
Self-rated confidence			x			x	x		
Knowledge test			x			x	x		
Practice change						x	x		
Intention to change practice						x	x		
Economic									x

*VLE* virtual learning environment

### Participants

#### Study setting

This study will be conducted virtually using Queen Mary University of London (QMUL) virtual learning environment (VLE), online questionnaires (SurveyMonkey), and Microsoft Teams, eradicating the need for participants to travel, reducing both cost and participants’ time. Participating clinicians will be working in a variety of settings (acute and community, special schools, both national health service and private settings) within the UK, where the data will be collected. Each participant’s data will be collected under their unique student number. To ensure anonymity, once data collection is complete, student numbers will be additionally coded.

#### Eligibility criteria

The aim of the inclusion and exclusion criteria is to ensure that participants are actively involved in assessment and treatment of ambulant CYPwCP and have currently available opportunities to apply the taught knowledge and skills in their workplace. The eligibility criteria were reviewed during the stakeholder focus groups including both clinicians and educators. Focus groups found inclusion and exclusion criteria appropriate for the feasibility trial (see Supplementary material).

Inclusion criteria are as follows:

- 18 years of age or older
- Physiotherapists currently providing assessment and treatment to ambulant children and young people with cerebral palsy
- Practicing within the UK (any National Health Service or private practice setting)

Exclusion criteria are as follows:

- Outside of UK
- Not currently employed as physiotherapist or on a career break
- In rotational posts, where they could rotate to specialty not managing ambulant CYPwCP

### Intervention

#### Design and refinement

This educational intervention uses a multicomponent approach grounded in behavioural change techniques (BCTs). The overall aim of the intervention is to improve gait-related clinical practice.

**Fig. 2:**
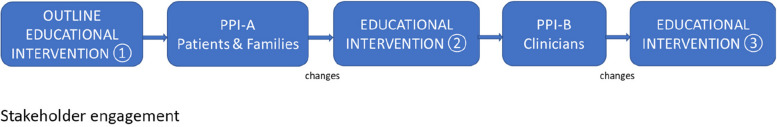
Stakeholder engagement

Intervention (VGAPP) will be delivered via QMUL VLE and will comprise of pre-course resources and a 6-week course. Content of the VGAPP course has been developed based on evidence from the scientific literature, current best practice and informed by the scoping review [17], qualitative study [15] and results from a national survey of paediatric physiotherapists in the UK (unpublished, in review). Stakeholder engagement has been integral to the research and intervention design, delivery and evaluation process and included Patient and Families (PPI-A) interviews and Clinicians and Educators Focus Groups (PPI-B) (Fig. 2). PPI-A included children, young people and their families who have a lived experience of cerebral palsy and received IGA as part of management of their condition. PPI-A was involved in the design of intervention prior to involving clinicians in order to ensure that the project is centred around the needs of patients and to ensure that the practice behaviour change, and transfer of knowledge will directly benefit patients and their families. Themes, subthemes and illustrative quotes from patients and parents’ interviews and changes applied to the intervention and evaluation content are available in the Supplementary Table 1. PPI-B were representatives from all UK nations, with a variety of paediatric physiotherapy specialisms, experience levels and from different work settings, thus providing invaluable insight and the opportunity for further refinement of the intervention design (in the areas of recruitment, eligibility criteria, sample size, control group intervention), content, delivery and evaluation methods. Themes, subthemes, and illustrative quotes from clinicians and educators focus groups and changes applied to intervention and evaluation content are available in the Supplementary Table 2.

Through this process, several changes were implemented to the intervention content and assessment process in areas of communication, patient/family perspectives, orthotics, and linking elements of gait-related practice to the ICF domains. A detailed PPI involvement report, including the educational intervention refinement process is available from the corresponding author on reasonable request.

#### Pre-intervention resources

Pre-intervention resources will include the pre-course manual, ‘meet and greet’ forum and the reading list. Participants will be able to complete a self-diagnostic tool to identify and reflect on their current IGA engagement and barriers to confident gait-related practice.

#### Intervention components

The intervention will be a stand-alone, post-graduate level educational event delivered fully on-line. It will employ the delivery of weekly on-line plenary sessions incorporating active learning — synchronous on-line problem-based learning sessions and seminars integrating elements of experimental learning within the learning community. These sessions will be delivered by experienced educators and clinicians working in the instrumented gait analysis laboratories, with a track record of delivering education within the field of gait analysis and paediatric neurodisability. Educators will be approached via email by the lead researcher. Content of the intervention will encompass an array of gait analysis methods and an overview of equipment currently used in the clinical practice. This will include but will not be limited to clinical outcome measures, measurement software, videography techniques and setup, 3-dimensional motion laboratory equipment, and laboratory setup (examples of Vicon and Codamotion Systems). Intervention will comprise of weekly tasks (asynchronous) to facilitate revision and application in practice and formative assessment/feedback opportunities (short knowledge quizzes, open questions within the discussion forum) to support learning autonomy and facilitate participant’s recall and self-regulation. Table 2 provides an indicative number of hours for each activity to give an overall picture of the workload a participant would be expected to undertake.

**Table 2:** Intervention learning format and an indicative number of hours for each activity

Learning format	Module-specific breakdown	Hours
**Content**	Seminars/PBL sessions	5
	Lectures/plenary	13
	On-line live forum interaction	2
**Student/peer learning**	Discussion/group work	4
**Student independent learning time**	Pre-session preparations (total)	4
	Completing assessments (incl. formative)	3
**Total study hours (6 weeks total)**		**31**
**Pre-course preparation**		2–4

The intended learning outcomes (ILOs) have been designed and benchmarked against the QAA Statements Physiotherapy (2001) Academic Content.

Academic content is as follows:

1. Demonstrates an understanding of the interdisciplinary knowledge that underpins gait analysis practice including elements of human anatomy, biomechanics, and gross motor development: C1
2. Demonstrates an understanding of the principles of typical gait pattern and how movement patterns are likely to be affected by some of the childhood diseases: C1
3. Demonstrates an understanding of the available measurement technologies and the principles on which they are based: C1

Disciplinary skills are as follows:

4. Applies variety of gait assessment methods in context of own practice and service delivery: B1 and C2
5. Uses the gait analysis outputs in clinical practice to aid treatment decision-making and measurement — in line with clinical reasoning paradigms and evidence-based practice: A1, B1, and C2
6. Communicates assessment findings and gait-related decision-making effectively with multidisciplinary team, patients, and families: A2, A3, B2, and C2

Attributes are as follows:

7. Cultivates an individualised, patient-centred approach to assessment and treatment planning: B2
8. Reflects on own practice to identify the needs within own role and wider aspects of service delivery: A3, A4, and B2 (health and social care equivalent B4)
9. Demonstrates a creative drive to implement the knowledge and skills, improve own practice, and support development of others: A3, A4, and B2 (health and social care equivalent B3)

### Behaviour change techniques (BCT)

Utilisation of the BCT taxonomy [20] will support refinement of the targeted behaviours. It will also support the process evaluation analysis to gain understanding of how the change is expected to take place [21] and related barriers and facilitators of implementing the feasibility trial. To support knowledge transfer, several behaviour change techniques will be used in the intervention content.

Prior to the course, participants will gain access to a diagnostic session to identify potential internal and/or external barriers to their gait-related practice. They will be encouraged to set their personal and service goals and will be supported in making plans for delivery. Participants will share their plans and progress as part of the evaluation process.

A variety of synchronous (problem-based learning sessions) and asynchronous resources (lectures, reading links and podcasts) will incorporate instruction on how to perform new or refined gait-related practice behaviours. These resources will also support shaping of the participant’s knowledge through instruction and demonstration on how to perform the behaviours and setting clinically oriented practical tasks focusing on the behaviour. Throughout the course, participants will be provided strategies to support behaviours through associations such as regular prompts and cues, ideas on restructuring of their clinical environment to improve their gait assessment quality and techniques, or through objects which could be added into their environment (such as outcome measure templates — digital and/or printed). A virtual learning community, created through group chats and discussion forums, will aim to support emergence of the identity associated with changed behaviours.

**Fig. 3:**
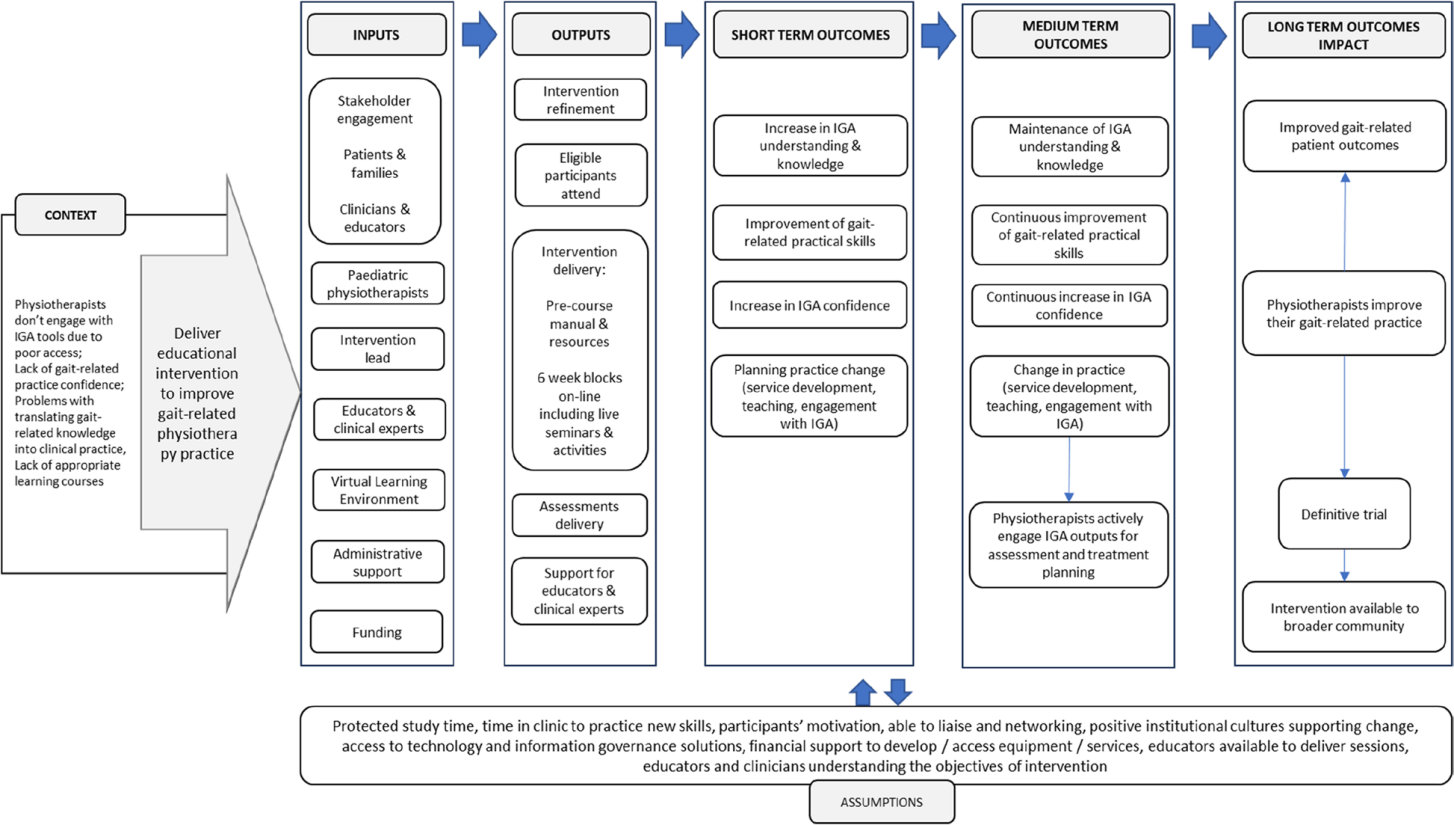
Feasibility RCT study logic model

Figure 3 outlines the simplified logic model of the study.

#### Control group intervention

To compare the effects of the intervention against usual practice, participants allocated into the control group will be asked to continue with their usual practice. At the point of enrolment, the control group will gain access to the virtual learning environment and receive basic orientation resources, but no training or guidance will be offered during this time. Participants in the control group will be asked to complete the same measurements as those in the intervention group and at the same timepoints (Table 1). The control group will be offered the full intervention after the completion of the third round of assessments. Provision of educational content and its timing in the control group were reviewed during the stakeholder focus groups including of clinicians and educators.

#### Outcome measures

Outcome measures were grouped as primary outcome measures (trial feasibility-related outcomes) and secondary outcome measures (trial research-related outcomes) collated in Table 3.

**Table 3:** Summary of primary and secondary outcome measures, hypotheses and analysis methods planned in the study

Variable/task	Hypothesis/target	Outcome measure	Method of analysis
***Primary***			
Recruitment	Study will recruit 24 participants	Number of participants	Descriptive statistics
Retention	Study will retain 75% of sample	Number of participants	Descriptive statistics
Engagement	The average proportion of completed VLE items will be ≥ 66%	Average proportion of completed VLE items	Descriptive statistics
***Secondary***			
Knowledge	EG will have improved knowledge scores relative to CG EG will increase knowledge scores EG will maintain knowledge scores	MCQ test	Effect sizes and 95% CIs from repeated measures ANOVA test and Kruskal–Wallis *H*-test
Skills	EG will have improved practical skills scores relative to CG EG will increase practical skills scores EG will maintain practical skills scores	OSCE	Effect sizes and 95% CIs from repeated measures ANOVA test and Kruskal–Wallis *H*-test
Attitudes	EG will have improved gait-related practice behaviours	Questionnaires: Self-reported knowledge and confidence, practice change, practice change planning, beliefs, semi-structured interviews	Effect sizes and 95% CIs from repeated measures ANOVA test Framework method for qualitative data (Ritchie 2014)
Satisfaction	EG will demonstrate a high satisfaction rate	Satisfaction questionnaire	Descriptive statistics

*EG* experimental group, *CG* control group, *VLE* virtual learning environment, *MCQ* multiple-choice question, *OSCE* observed standardised clinical examination

Recruitment will be determined as feasible if study is able to recruit 24 participants within 4 months [22, 23]. Retention rates will be considered at two stages: (1) from expression of interest to consent — it will be deemed feasible if greater than 50%, and (2) from consent to course completion — it will be deemed feasible if greater than 75% [24, 25]. Additionally, engagement (participants’ interactions with an online system) data will be collected during intervention via the analytics tools in the QMUL Virtual Learning Environment which log the detail of activity access, time, and completion for each component. These analytic tools are part of the general-purpose dashboard and provide an algorithmic representation of student online behaviours based on whether the behaviour occurred and for how long, rather than quality of these behaviours. Previous studies show that these analytics have been positively correlated with student performance [26–28]. It will be deemed feasible if the average proportion of completed learning sessions and tasks will be ≥ 66%.

Secondary outcomes are as follows: knowledge, skills, attitudes, and satisfaction will be collected via online questionnaires (SurveyMonkey) and during skill tests (OSCE). Knowledge, skills, and attitudes will be collected at three timepoints (Table 1).

##### Baseline (pre-intervention)

- Questionnaire including background (demographics, current gait analysis practice, access to IGA equipment, barriers to gait analysis practice), attitudes (reasons for joining the study, anticipated changes in practice after the intervention, beliefs), confidence (self-rated), and knowledge (self-rated and multiple-choice question test)
- Objective structured clinical examination (OSCE) of a patient case: Assessment will be delivered on-line, recorded and scored against a standardised scoring sheet including gait-related clinical reasoning and treatment planning based on evidence and findings, problem-solving, systematicity of approach, ability to link various types of gait-related information, confidence in engagement with gait data, analysis of gait graphs, communication (including use of gait-related terminology, providing lay explanations to a parent), and implementation of biopsychosocial model or ICF to decision-making

##### Post intervention (immediately after 6-week intervention)

- Questionnaire including attitudes (planning practice change, implemented practice change, beliefs), confidence (self-rated), knowledge (self-rated and multiple-choice question test), and satisfaction (experimental group only)
- OSCE of a different patient case (scored against the same criteria as at baseline)

##### Re-test (4-month post-intervention)

- Questionnaire attitudes (planning practice change, implemented practice change, beliefs), confidence (self-rated), and knowledge (self-rated and multiple-choice question test)
- OSCE of a different patient case (scored against the same criteria as at baseline)

Knowledge and skills retention as well as attitudes will be measured between timepoints, with a focus on changes between baseline and immediately post intervention and at 4-month follow-up. Satisfaction questionnaire will contain 28 items, each assessed on a 5-point Likert scale, related to the relevance and scientific quality of the content, the educational structure, and delivery. Satisfaction feedback will be collected immediately after intervention delivery (experimental group).

### Sample size

Considering the study objectives and recommendations, the target sample size will be of a minimum 12 participants per trial arm; therefore, a minimum of 24 in total is anticipated. Guidance from the National Institute for Health Research (NIHR) indicates that a sample size of 30 is appropriate to answer the questions posed by a feasibility trial [23]. A lower number of participants will be better suited for an educational intervention for clinicians — it will ensure delivery of a high-quality learning experience and allow for active engagement with tutors during problem-based learning within the experimental group. Furthermore, the stakeholder focus groups including of clinicians and educators reviewed the proposed sample size and reported it as appropriate for the feasibility trial.

### Recruitment

Participants will be recruited via the largest national paediatric physiotherapy network (Association of Chartered Paediatric Physiotherapists) using bulletins, social media, and targeted emails to team leads across the UK. The advertisement will provide general information about the intervention and the research study together with inclusion and exclusion criteria. Upon expression of interest, participants will be screened against eligibility criteria, and the participant’s information sheet and consent form will be sent to prospective participants via email. Participants will return signed consent forms electronically to the research lead. In line with advice from the Clinician and Educator Focus Group (PPI-B), the recruitment of study participants will commence early to ensure that participants are able to make suitable arrangements in the workplace, such as request study leave and ‘block time’ to attend synchronous sessions etc.

#### Participant timeline

Time schedule of enrolment, intervention, and assessments is presented in Table 1. After the eligibility criteria screen and receipt of their written informed consent, 24 participants will be enrolled to the study. After random allocation to the trial arms, participants will receive access to the password-protected online platform hosted by Queen Mary University of London. All participants will be asked to complete the baseline assessment including the questionnaire (background, attitudes, knowledge, and skills) and objective structured clinical examination (OSCE) of a patient case (assessment will be delivered on-line, recorded, and scored against standardised scoring sheet). After completing the baseline assessment, participants assigned to the experimental arm will gain access to the pre-course learning resources (6 weeks prior to start of the course). The experimental group will commence the 6 week blocks of intervention including pre-recorded resources, problem-based learning tasks, discussion forums, and live sessions. At 6 weeks, participants from both arms will be asked to complete the second assessment including the questionnaire (attitudes, knowledge and skills, and satisfaction scores in experimental group only) and the second OSCE of a patient case. Four months after the intervention, participants in both trial arms will be asked to complete the third assessment including the questionnaire (attitudes, knowledge, and skills) and the OSCE of a patient case. Once all the data is collected, participants in the control group will gain access to the prereading resources and start the 6-weekly intervention sessions.

### Assignment of intervention

#### Allocation, concealment mechanism, and implementation

Participants who meet the inclusion criteria and return the consent form will be assigned an ID number in the Microsoft Excel spreadsheet. Participants will be assigned to groups randomly. In case there are more eligible physiotherapists than spaces, participants will be chosen by the number generation software which will be used in the allocation process. This will be conducted by an external person not related to the study or the research team. To avoid contamination, participants from the same healthcare trusts will be randomised to the same group.

Information about group randomisation will be provided in the participant’s information sheet. Participants in this study will not be blinded to the group allocation or deceived. This was discussed in the stakeholder focus groups who agreed that in the context of clinical practice, deceiving participants could mean a loss of their study/annual leave if pre-booked specifically to attend the intervention as well as potential cancelations of clinics in the control group. Participants will be informed about their allocation at the time of receiving instructions with the QMUL VLE platform access. At this time, the control group will be informed about timings of gaining their access to the full intervention and all resources provided to the experimental group after final assessments are completed. Participants will be informed that they are free to withdraw at any time without needing to provide a reason and with no penalties or detrimental effects.

### Data collection, management, and analysis

In line with accepted practice for feasibility studies, no power analysis will be conducted, and all analyses will be exploratory only [29]. Data analysis will be performed after the last trial participant has completed final assessments (outcomes at 4 months post intervention). Data will be managed initially in Microsoft Excel software and analysed using IBM SPSS statistics software. Table 3 provides a summary of outcome measures, hypotheses, and analysis planned in the study.

#### Data management and research governance

A baseline table (descriptive statistics and frequencies) will compare the demographic and clinical characteristics including gender, age, experience, education, practice setting, contract type, study leave availability to participate in intervention, access to equipment, and gait analysis training. The primary outcomes will be reported using descriptive statistics. The quantitative variables will be presented as means and standard deviations.

A preliminary analysis of between-group differences will be conducted to determine the range of potential effect sizes from repeated measures ANOVA. Feasibility outcomes will be presented as number of participants meeting the a priori definitions. Kendall’s tau-b (*τ*_b_) correlation coefficient will be used to measure of the strength and direction of association that exists between two variables measured on at least an ordinal scale. To explore the extent and patterns of missing outcome data, we will report the proportion of missing values per item and the proportion of participants who will complete all items on the questionnaires. The proportion of missing data will also be reported for the other key outcomes and compared between the participants from intervention and control groups.

Qualitative data will be analysed according to the framework approach [30], a realist approach located within an interpretivist frame. The opinions and experiences of participants will be explored to understand any barriers and facilitators related to running of the educational intervention. During active familiarisation, the textual data will be coded, and codes will be organised into themes and subthemes to construct a thematic framework to aid indexing. To ensure rigour and consistency, the analysis process will undergo investigator triangulation. In this process, different observers, examiners, and analysts will compare and check data collection and/or interpretation [30, 31]. Qualitative data will be presented as quotes and descriptive summaries.

### Process evaluation and implementation outcomes

The process evaluation has been informed by Medical Research Council guidance on process evaluation of complex interventions [32, 33] and the Implementation Outcome Framework (IOF) [34]. Proctor et al. described eight implementation outcomes in the IOF: acceptability, adoption, appropriateness, feasibility, fidelity, implementation cost, penetration (or coverage), and sustainability. Each of these implementation outcomes aligns with important considerations for trial design and implementation; however, the ‘adoption’ outcome does not directly align with process evaluation of our current feasibility trial design and delivery, as it is not offered by other educational providers. Therefore, seven out of eight implementation outcomes will be included in this process evaluation. Acceptability of the intervention and of the assessments will include data on the duration, content, and delivery methods (including satisfaction scores). Synthesis of satisfaction scores, feedback, and reports on participants’ logistics related to taking part in the trial (protected study time, ensuring opportunities in practice, assessment burden) will be carried out. The findings will be supplemented with observations made by the researchers, educators, administrative staff, and examiners throughout the implementation of the intervention. Collectively, these will provide information on the acceptability of the trial measurements and the intervention. Feasibility measures will include participant recruitment rate, retention, and engagement thresholds as described in the ‘Methods’ section. The process evaluation will include analysis of proportion of eligible participants being offered trial and, if possible, proportion of participants in the population represented by eligibility criteria (coverage).

Baseline comparisons will be conducted to detect any substantial differences between participants recruited from the control and intervention arms. Sample size and anticipated effect size defined for the definitive trial will be reviewed and assessed for feasibility. Participant withdrawals and number of participants lost to follow-up (and where possible reasons and participants’ key baseline characteristics) will be analysed. The study protocol adherence will be reviewed within the research team. Fidelity to the trial protocol including follow-up, dosage of the intervention, crossover between study arms, and adherence to intervention delivery plan will be assessed against study protocol and participant timelines. Any changes to the protocol will be reported.

Furthermore, appropriateness of the trial design for the trial aim, inclusion and exclusion criteria, outcome measures, and intervention components will undergo an exploratory analysis of participants’ outcomes, engagement with content, and assessments, together with qualitative analysis of participants and educators’ feedback. Sustained participant interest throughout the trial period and sustained staffing levels to deliver and facilitate participants’ learning journey during intervention will be explored to inform the sustainability criteria for the definitive trial. The implementation cost analysis will be explored with the aim to inform the design of a full cost-utility analysis alongside a future definitive trial. Implementation cost will include the cost of administration involved in running the trial and cost related to production and delivery of the intervention and assessment components — such as speaker fees, and OSCE examiners and moderators will be reviewed.

In addition, the COM-B model and the behaviour change techniques taxonomy (BCTT) [35], widely used frameworks in behaviour change and implementation research, will support the process evaluation analysis and an in-depth exploration of the barriers and facilitators of implementing the feasibility trial.

## Discussion

This article describes the protocol of a study evaluating the feasibility of conducting definitive RCT of the educational intervention for paediatric physiotherapists working with ambulant CYPwCP in the UK. This feasibility study was designed to assess predefined criteria related to the evaluation design (such as reducing uncertainty around recruitment, retention, choice of outcomes, analysis) and the intervention (its content and delivery, acceptability, adherence, cost-effectiveness, etc.) in line with the current guidance [32, 33].

The educational intervention planned for this trial intends to integrate the complexity of knowledge, skills within the realities of clinicians’ practice to support knowledge translation to influence the practice behaviour change. Due to its complexity, the design of the study was preceded by in-depth research studies of the intervention’s context and implementation factors within the clinical practice reality of paediatric physiotherapists. This included close collaboration with stakeholders — patients and their families, clinicians, and clinical educators [33].

The need for gait analysis training was clearly identified in previous study of physiotherapists in the UK [13]. Despite extensive gait-related practice [36, 37], evidence of how paediatric physiotherapists engage with instrumentation or access the IGA training is sparse. There are currently many gait-related courses available world-wide delivered by a variety of providers specifically targeting this clinical group (CMAS workshop 2023). Although there is a rich training offer, the impact of training on skills and behaviour, evaluation of needs, and barriers to knowledge transfer are not addressed in the current literature showing an evidence gap (CMAS 2023 education workshop). The impact of existing educational interventions is rarely reported [38, 39] and concerns low levels of evaluation evidence, omitting evaluation clinical behaviour change or organisational impact. Our previous studies show that transfer of gait-related knowledge from the classroom to the clinic room also poses challenges to clinicians at different levels of practice expertise [15]. The lack of institutional resources (financial, such as availability of funding for staff’s training or limited study leave), spatial and temporal to promote implementation of new procedural skills and motivation to engage with learning, may also influence low uptake of professional training.

One of the main challenges will be associated with possible low uptake in the study and high drop-out rate. High work pressures and limited time to study may result in reduced opportunity or willingness to participate in the intervention and multiple assessments.

### Limitations

Participants in this trial will not be blinded to allocation. After discussions within the research team and stakeholder focus groups, it was decided that if a participant secures study leave to take part in the 6-week intervention (potentially taking time off clinical work which may lead to cancellation of clinics) and would not receive the intervention due to allocation to the control group — this may result in loss of study leave and could have a potentially negative impact on the patient’s care by added waiting time.

The intervention lead is a paediatric physiotherapist experienced in gait-related practice which may be a source of potential bias. To mitigate this risk, multiple educators and clinical experts will be appointed to co-deliver the intervention, and additional examiners and moderators will be blinded to participants’ allocation. The intervention lead will keep a reflective diary and will have access to de-brief meetings within the research team [40]. Involvement of a considerable number of experts co-delivering the content of the intervention may pose risk to intervention integrity. To mitigate this risk, the intervention lead will be providing detailed 1:1 briefing about the study, targeted behaviours, session aims, and ILOs.

### Generalisability

A relatively small sample planned for this feasibility study may pose questions regarding the applicability of findings to the future definitive trial and other studies. To ensure that the feasibility sample is representative of the UK paediatric physiotherapists, the study will be broadly advertised to reach therapists in all four UK countries and across the healthcare sectors.

Despite the extensive context research, a wide array of primary and secondary outcome measures planned to be used in the process evaluation, there may be factors influential to the trial but not be captured by the feasibility testing. Use of MRC guidance on process evaluation of complex interventions [32, 33] and the IOF [34] will ensure thorough investigation of the change mechanisms and how the effects will occur [32, 41]. Furthermore, the COM-B model and BCTT [35] are useful tools to characterise the targeted behaviours and content of educational interventions focused on continuing professional development in healthcare [42]. These were used throughout design of the study and will support the process evaluation to further advance understanding of their mechanisms of action.

With the detailed planning of this protocol and careful consideration of challenges and limitations, this study will offer essential preliminary data about the feasibility of implementing the VGAPP intervention to improve gait-related practice of paediatric physiotherapists in the UK. Study findings will provide a comprehensive understanding of whether a full randomised control trial is viable and identify any areas which could be enhanced. Furthermore, this study will contribute to a body of educational research into clinical training of healthcare professionals and IGA training.

### Supplementary Information

**Supplementary Material 1.**

**Supplementary Material 2.**

## Data Availability

Data sharing is not applicable to this article as no datasets were generated or analysed during the current study.

## Abbreviations

BCTT: Behaviour change techniques taxonomy
CMAS: Clinical Movement Analysis Society
COM-B: Capability, opportunity, motivation, behaviour model
CONSORT-PF: Consolidated Standards of Reporting Trials for Pilot and Feasibility trials
CYPwCP: Children and young people with cerebral palsy
ICF: International Classification of Functioning, Disability and Health
IGA: Instrumented gait analysis
ILO: Intended learning outcomes
IOF: Implementation Outcome Framework
MRC: Medical Research Council
NICE: National Institute for Clinical Excellence
OSCE: Objective structured clinical examination
PPI: Patient and public involvement
SPIRIT: Standard Protocol Items: Recommendations for Interventional Trials
VGAPP: Virtual gait analysis course for paediatric physiotherapists
VLE: Virtual learning environment

## References

[1] NICE. Clinical guideline [CG145] Spasticity in under 19s: management. https://www.nice.org.uk/guidance/cg145/chapter/1-guidance2016.

[2] Wren TA, Lening C, Rethlefsen SA, Kay RM (2013). Impact of gait analysis on correction of excessive hip internal rotation in ambulatory children with cerebral palsy: a randomized controlled trial. Dev Med Child Neurol.

[3] Theologis T, Stebbins J (2010). The use of gait analysis in the treatment of pediatric foot and ankle disorders. Foot Ankle Clin.

[4] Kay RM, Wren TA, Bowen RE, Otsuka NY, Scaduto AA, Chan LS (2009). Influence of gait analysis on decision-making for lower extremity surgery. Dev Med Child Neurol.

[5] Lofterod B, Terjesen T, Skaaret I, Huse AB, Jahnsen R (2007). Preoperative gait analysis has a substantial effect on orthopedic decision making in children with cerebral palsy - comparison between clinical evaluation and gait analysis in 60 patients. Acta Orthop.

[6] Franki I, De Cat J, Deschepper E, Molenaers G, Desloovere K, Himpens E (2014). A clinical decision framework for the identification of main problems and treatment goals for ambulant children with bilateral spastic cerebral palsy. Res Dev Disabil.

[7] Rasmussen HM, Pedersen NW, Overgaard S, Hansen LK, Dunkhase-Heinl U, Petkov Y (2019). Gait analysis for individually tailored interdisciplinary interventions in children with cerebral palsy: a randomized controlled trial. Dev Med Child Neurol.

[8] McGinley J, Dobson F, Ganeshalingham R, Shore B, Rutz E, Graham HK (2012). A systematic review of single event multilevel surgery for children with cerebral palsy. Dev Med Child Neurol.

[9] Wren T, Otsuka N, Bowen R, Scaduto A, Chan L, Dennis S (2013). Outcomes of lower extremity orthopaedic surgery in ambulatory children with cerebral palsy with and without gait analysis: results of a randomised controlled trial. Gait Posture.

[10] Gough M, Shortland AP (2008). Can clinical gait analysis guide the management of ambulant children with bilateral spastic cerebral palsy?. J Pediatr Orthop.

[11] Chang FM, Seidl AJ, Muthusamy K, Meininger AK, Carollo JJ (2006). Effectiveness of instrumented gait analysis in children with cerebral palsy–comparison of outcomes. J Pediatr Orthop.

[12] Gaston MS (2019). CPIPS: musculoskeletal and hip surveillance for children with cerebral palsy. Paediatr Child Health.

[13] Toro B, Nester C, Farren P (2003). The status of gait assessment among physiotherapists in the United Kingdom. Arch Phys Med Rehab.

[14] Baker R, Esquenazi A, Benedetti MG, Desloovere K (2016). Gait analysis: clinical facts. Eur J Phys Rehabil Med.

[15] Hebda-Boon A, Zhang B, Amankwah A, Shortland AP, Morrissey D (2022). Clinicians’ experiences of instrumented gait analysis in management of patients with cerebral palsy: a qualitative study. Phys Occup Ther Pediatr..

[16] Wren TA, Elihu KJ, Mansour S, Rethlefsen SA, Ryan DD, Smith ML (2013). Differences in implementation of gait analysis recommendations based on affiliation with a gait laboratory. Gait Posture.

[17] Hebda-Boon A, Tan XL, Tillmann R, Shortland AP, Firth GB, Morrissey D (2023). The impact of instrumented gait analysis on decision-making in the interprofessional management of cerebral palsy: a scoping review. Eur J Paediatr Neurol.

[18] Chan AW, Tetzlaff JM, Altman DG, Dickersin K, Moher D (2013). SPIRIT 2013: new guidance for content of clinical trial protocols. Lancet.

[19] Eldridge SM, Chan CL, Campbell MJ, Bond CM, Hopewell S, Thabane L (2016). CONSORT 2010 statement: extension to randomised pilot and feasibility trials. Pilot Feasibil Stud.

[20] Michie S, Richardson M, Johnston M, Abraham C, Francis J, Hardeman W (2013). The behavior change technique taxonomy (v1) of 93 hierarchically clustered techniques: building an international consensus for the reporting of behavior change interventions. Ann Behav Med.

[21] Campbell M, Fitzpatrick R, Haines A, Kinmonth AL, Sandercock P, Spiegelhalter D (2000). Framework for design and evaluation of complex interventions to improve health. BMJ.

[22] Jacques RM, Ahmed R, Harper J, Ranjan A, Saeed I, Simpson RM (2022). Recruitment, consent and retention of participants in randomised controlled trials: a review of trials published in the National Institute for Health Research (NIHR) Journals Library (1997–2020). BMJ Open.

[23] Lancaster GA, Dodd S, Williamson PR (2004). Design and analysis of pilot studies: recommendations for good practice. J Eval Clin Pract.

[24] Trivedi RB, Szarka JG, Beaver K, Brousseau K, Nevins E, Yancy WS (2013). Recruitment and retention rates in behavioral trials involving patients and a support person: a systematic review. Contemp Clin Trials.

[25] Poongothai S, Anjana RM, Aarthy R, Unnikrishnan R, Narayan KMV, Ali MK (2023). Strategies for participant retention in long term clinical trials: a participant -centric approaches. Perspect Clin Res.

[26] Jayaprakash SM, Moody EW, Lauría EJM, Regan JR, Baron JD (2014). Early alert of academically at-risk students: an open source analytics initiative. J Learn Analytics.

[27] Macfadyen L, Dawson S. Numbers are not enough. Why e-learning analytics failed to inform an institutional strategic plan. Educational Technology & Society. 2012;15(3):149–63.

[28] Atif A Froissard C, Liu DY, Richards D. Validating the effectiveness of the moodle engagement analytics plugin to predict student academic performance. Americas Conference on Information Systems. in 21st Americas Conference on Information Systems, AMCIS 2015 (pp 1-10).

[29] Teresi JA, Yu X, Stewart AL, Hays RD (2022). Guidelines for designing and evaluating feasibility pilot studies. Med Care.

[30] Ritchie J (2014). Qualitative research practice: a guide for social science students and researchers.

[31] Elliott R, Fischer CT, Rennie DL (1999). Evolving guidelines for publication of qualitative research studies in psychology and related fields. Br J Clin Psychol.

[32] Moore GF, Audrey S, Barker M, Bond L, Bonell C, Hardeman W (2015). Process evaluation of complex interventions: medical research council guidance. BMJ.

[33] Skivington K, Matthews L, Simpson SA, Craig P, Baird J, Blazeby JM (2021). Framework for the development and evaluation of complex interventions: gap analysis, workshop and consultation-informed update. Health Technol Assess.

[34] Proctor E, Silmere H, Raghavan R, Hovmand P, Aarons G, Bunger A (2011). Outcomes for implementation research: conceptual distinctions, measurement challenges, and research agenda. Adm Policy Ment Health.

[35] Michie S, Atkins L, West R (2014). The behaviour change wheel.

[36] Franki I, Desloovere K, De Cat J, Feys H, Molenaers G, Calders P (2012). The evidence-base for basic physical therapy techniques targeting lower limb function in children with cerebral palsy: a systematic review using the international classification of functioning, disability and health as a conceptual framework. J Rehabil Med.

[37] Rapson R, Latour JM, Marsden J, Hughes H, Carter B (2022). Defining usual physiotherapy care in ambulant children with cerebral palsy in the United Kingdom: a mixed methods consensus study. Child Care Health Dev.

[38] Malone JB, Burns JD, Belthur MV, Karlen JW (2022). Motion laboratory gait analysis and orthopedic resident education: preliminary results. J Pediatr Orthop B.

[39] Baskwill A, Belli P, Keller L (2017). Evaluation of a gait assessment module using 3D Motion capture technology. Int J Ther Massage Bodywork.

[40] Korstjens I, Moser A (2018). Series: practical guidance to qualitative research. Part 4: Trustworthiness and publishing. Eur J Gen Pract.

[41] O'Cathain A, Croot L, Duncan E, Rousseau N, Sworn K, Turner KM (2019). Guidance on how to develop complex interventions to improve health and healthcare. BMJ Open.

[42] Konnyu KJ, McCleary N, Presseau J, Ivers NM, Grimshaw JM (2020). Behavior change techniques in continuing professional development. J Contin Educ Health Prof.

