# Supplementary material for: Can on-line gait training improve clinical practice? Study protocol for feasibility randomised controlled trial of an on-line educational intervention to improve clinician’s gait-related decision-making in ambulant children and young people with cerebral palsy
Source: Pilot Feasibility Stud. 2024 May 14;10:76. doi: 10.1186/s40814-024-01477-5 (PMC11091998; doi:10.1186/s40814-024-01477-5)
Supplement: Supplementary file 1 — Supplementary Material 1. [file 40814_2024_1477_MOESM1_ESM.docx]

Supplementary Table 1. Themes, subthemes and illustrative quotes from patients and parents’ interviews, and changes applied to the intervention and evaluation content. IGA – instrumented gait analysis, ICF - International Classification of Functioning, Disability and Health.

| **Theme1: EXPERIENCE OF GAIT ANALYSIS** | | | | |
| --- | --- | --- | --- | --- |
| **Subtheme from parents** | **Quote from parents** | **Actions taken** | **Quote from children** | **Subtheme from children** |
| **Positive but lacking information** | *When I did go in, I found it quite fascinating, and I don’t think it was what I expected. I didn’t ask a lot of questions before as I have a lot of trust in the team.’ Parent3* | Intervention content added / refined:   - Asynchronous sessions highlighting patients’ views on IGA processes. - Tasks related to discussing IGA with children and families in lay language. - Podcasts with expert in the field of neurodisability discussing gait-related goal setting, expectations, participation & revision of role of the biopsychosocial models in the gait-related rehabilitation. | ‘Overall, very positive experience, I can’t fault it.’ Young Person 1 | **Positive but lacking information** |
|  | *‘First time round he was not very cooperative, and he needed a lot of encouragement to get him what they want him to do. But the second time he was fine. Parent1* |  | *‘For the first time when I went, I didn’t have a clue what it (gait analysis) was.’ Child1* |  |
|  | *When she was referred, I don’t think the explanation was good, so I went and researched […] the person who ran the gait analysis – he was great with (my child), he was explaining, made her laugh, made it fun.’ Parent4* |  |  |  |
|  | *the extent of the procedure and its importance in management builds up a level of anxiety and pressure in the patient, for the child. […] It's very fatiguing.’ Parent2* |  |  |  |
| **Stressful test** | *‘I think the thing to take on board is that it can leave young people feeling quite deflated. […] My child felt like they failed the test.’ Parent4* |  | *I knew why I was going there – to see if I should have an operation. […] there is a bit of stress in those things... Young Person1* | **Stressful test** |
|  | *‘It gets very demoralizing, when you are being asked to perform series of walks like that and very intense and nothing changes after.’ Parent2* |  |  |  |
| **Theme2: COMMUNICATION** | | | | |
| **Subtheme from parents** | **Quote from parents** | **Actions taken** | **Quote from children** | **Subtheme from children** |
| **Communication with patients & families** | *‘If you would ask me, you have to teach better those people, you know, they have to communicate better to parents.’ Parent 1* | Intervention content added / refined:   - Guidance and support in networking and linking Participants with their local gait analysis services, - Asynchronous session on importance of communication pre- and post-IGA. - Tasks related to discussing IGA with children and families in lay language.   Evaluation content added / refined:   - OSCE marking criteria adjusted to give scores in areas of patient-centredness, goal setting and treatment planning with consideration of activity and participation domains of ICF, use of lay language when explaining IGA reports. - Questionnaire exploring attitude and clinical practice in patient-centred management and Multiple-Choice Knowledge Questions assessing understanding of the ICF. | *‘I would like to know what it means in terms of my results, what should I expect – I was like tell me so I can understand.’ Young person 2* | **Communication with patients & families** |
|  | *‘The report is complex; it gives you really good insight. It should be at the centre of the conversation but let’s speak in the layman terms and make parents part of the team. Do not make parents inadequate in this conversation.’ Parent 4* |  |  |  |
|  | *‘Sometimes parents are not very fluent in English language. we go home and we think about it. What happened? What did that mean? And you try to put all information together. I’m talking from my experience.’ Parent 1* |  |  |  |
|  | *‘With gait analysis it’s so important how we talk to these young people about something that it’s going to be their life.’ Parent 4* |  |  |  |
|  | *He (my child) is now big boy, and he knows what he needs to do, so if you communicate with him also what to do, we can follow that at home.’ Parent 1* |  |  |  |
| **Communication between professionals** | *‘Local physios and tertiary ones need to talk to each other, make a plan and so everyone is on the same page.’ Parent 4* |  |  | **Not applicable** |
|  | *‘We have never discussed the gait report with the physio. It seems like our physio has her own plan.’ Parent 2* |  |  |  |
|  | *I sometimes feel that nobody works together. It shouldn’t be like this. What if she (my child) didn’t have an advocate like me? Parent 4* |  |  |  |
|  | *Apparently, they are two different trusts, and they don’t have much communication at all. Parent 2* |  |  |  |
|  | *I think the reports were accessible to most physios, it’s just a matter of taking this additional interest in your patients and read the report. Parent2* |  |  |  |
