# Supplementary material for: Can on-line gait training improve clinical practice? Study protocol for feasibility randomised controlled trial of an on-line educational intervention to improve clinician’s gait-related decision-making in ambulant children and young people with cerebral palsy
Source: Pilot Feasibility Stud. 2024 May 14;10:76. doi: 10.1186/s40814-024-01477-5 (PMC11091998; doi:10.1186/s40814-024-01477-5)
Supplement: Supplementary file 2 — Supplementary Material 2. [file 40814_2024_1477_MOESM2_ESM.docx]

Supplementary Table 2. Themes, subthemes and illustrative quotes from clinicians and educators focus groups 1 and 2, and changes applied to intervention and evaluation content. IGA – instrumented gait analysis, CP – cerebral palsy.

| **Theme1: *THE STUDY DESIGN*** | | |
| --- | --- | --- |
| **Quote from PPI 1** | **Actions taken** | **Quote from PPI 2** |
| **Subtheme: Eligibility criteria** | | |
| *‘To make sure that the participant actually consolidated learning and have the experience of practicing what they've learned, you have to exclude the rotational posts.’* | Eligibility criteria remained unchanged. | *‘If they have the rotational component, they may not see kids with CP again for years.’* |
| *‘It's quite an open eligibility’* |  | *‘I think you want to be sure that you're going to have that follow up period. With them working in the right area.’* |
| **Subtheme: Recruitment** | | |
| *‘We run a gait course for the physios in the community […] and it takes a long time for people to get approval from managers.’* | Recruitment of study participants will commence early to ensure that there is enough time for participants to make suitable arrangements in the workplace. | *‘Learning should be factored into the study leave, otherwise you might get people doing this course in evenings and weekends and not actually getting the time that it needs to practice the skills.’* |
| **Subtheme: Sample size** | | |
| *‘I'm sure you're not going to struggle to find 24 participants’* | Sample size remained unchanged. | *‘I think 12 sounds reasonable because it's going to be huge analysis otherwise.’* |
| *‘You can get kind of 12 faces around the screen quite easily and pick up on body language and have opportunity to speak.’* |  | *‘You might have to adapt depending on, I suppose, things like what that might cost to run, how that might work out in terms of finance’* |
| *‘A much bigger group would be a much different experience for those who are involved in teaching and those who are participating’* |  |  |
| **Subtheme: Control arm** | | |
| *‘You know you're gonna get the teaching now or you're going to get it later. You're still going to get the teaching. I don't think that that's an issue.’* | Participants in the control group will gain access to the virtual learning environment and receive basic orientation resources.  control group will receive full intervention after the completion of third round of assessments. | *‘If they’re going to get the full course in October, they don’t need to be given anything in the first six weeks.’* |
| *‘I don't think during the experimental period they should receive anything’* |  | *‘I think you do need to give them a little bit of a ‘nugget’* |
| *‘Regardless of what you give them, […] they're already slightly likely to be more motivated individuals.’* |  | *‘I think if you give them the pre course reading then that's reasonable, but I think any more than that and you're not going to know whether the whole program is effective or not.’* |
| *‘Being given anything draws your focus so you will already be seeking out subconsciously when you see things.’* |  | *‘I think, as long as people are clear it might not be in the next three months. I think that's reasonable.’* |
| *‘If you're already motivated, bunch of people or if they already know each other, I don't think you can control them not getting in touch with each other on social media, catching up.’* |  |  |
| **Blinding of participants** | | |
| *‘If you're going to tell them whether this is the true intervention or if this is not the intervention you're measuring, then you've kind of influenced them a little bit before you've begun.’* | Participants will not be blinded or deceived to avoid loss of annual or study leave, and potential cancelations of clinics in the control group. | *‘I think I really wouldn't be fine with it if I was 50/50 to get it (intervention) or not, and I've taken all the study leave, gone through all the paperwork to get the study leave and all that… I think that would be problematic.’* |
| ***Theme 2: THE INTERVENTION DESIGN*** | | |
| **Quote from PPI 1** | **Actions taken** | **Quote from PPI 2** |
| **Subtheme: Content** | | |
| *‘I think it's fantastic and I think it's a really, really good introduction to gait analysis. I think all my team would love to be on it.’* | Content on orthotics prescription and decision-making will be added in the intervention weeks 4 and 5. | *‘I think it follows through in a really nice order.’* |
| *‘You need to have the principles yes, but you also need to see it in the context. You need to see it as part of the process. You need to be able to fit the pieces together.’* |  | *‘You've got all of your sort of physics stuff in that first week, and I just think you might find that helpful when you get to kinematics and kinetics, when you need to revisit some of those.’* |
| *‘I think having some flexibility between the weeks might be important.’* |  | *‘I do think there's something about the language […], you've got to use it to make it embedded in practice’* |
| *‘I think it would be very helpful to learn and understand the perspective of the child and family.’* |  | *‘I think it's also about making it easier to apply locally in a meaningful manner […] There’s a bit about the interpretation of the data and understanding what's coming back down.* |
| *‘Having an improved understanding of what an instrumented gait lab can do and what it can't do as well gonna improve the conversations that we have with families’* |  | *‘As part of your training, people need to learn to step away from being incredibly professional, step away from being clinical.* |
| *‘I suppose if they're bringing the cases, the discussions will just be around video analysis. They won't then have the opportunity for analysing graphs.’* |  | *‘I think you definitely have to have people prepare stuff […] when it's well facilitated and people have to come with something and to critique each other, I think that should be well.’* |
| *‘No matter how much reading material I've passed to people within my department, they don't read it, or they skim it. But when we sit down and we talk about it, that's where all the ideas come out. ‘* |  | *‘The other thing I just wondered, is in terms of orthotics. I didn't know it was there any kind of touching on that? I think you probably need orthotic assessment throughout, because it's part of your history taking when you meet the child with the CP’* |
| **Subtheme: Delivery & Virtual learning environment** | | |
| *‘I think asynchronous learning works really well. I've got experience with that being quite a good way of delivering* | Delivery style will remain unchanged. | *The benefit of the actual course is the learning environment* |
| *I think it's really exciting that it could be accessible to anyone in the country or internationally’* |  | *‘It (virtual learning) has become much more commonplace.’* |
| *we've all had to advance our digital skills for talking in remote groups and linking-in together and setting up different networks in the different way. So no, meeting face to face is not necessary.’ ‘* |  | *‘And it really comes back to learning styles, doesn't it? […] So, I think probably having a variety and means that you're going to at least get a greater breadth of coverage just froze or breath of learning.’* |
| *people can access it a lot more and they don't have to travel.* |  | *‘How you learn and how you're educated now is, I think, very different to how I was educated.’* |
| *‘Some of those methods that you've written down, allow that transfer (of knowledge) to happen.’* |  |  |
| ***Theme3: THE ASSESSMENTS OF THE COURSE*** | | |
| **Quote from PPI 1** | **Actions taken** | **Quote from PPI 2** |
| *‘I think we are all used to answering on-line questionnaires and providing feedback. But […] don’t make them too long and humorous. Focus and collect only data you need.’* | Assessment schedule and activities will remain unchanged. | *‘Have people explain to each other and having an observer - that's a really useful way of assessing. It’s really commonly used in teaching, coaching and stuff like that.’* |
| *‘Don't get lost in looking at gait analysis as a tool in its own right but looking at the efficacy of the teaching and how to teach gait analysis.’* |  | *‘Great idea on assessing their clinical reasoning in case studies. I have not seen it done is a non-university course.’* |
|  |  | *‘I think it's huge. I think you're undertaking a massive task; I think it’s amazing.’* |
